# Molecular determinant of the effects of hydrostatic pressure on protein folding stability

**DOI:** 10.1038/ncomms14561

**Published:** 2017-02-07

**Authors:** Calvin R. Chen, George I. Makhatadze

**Affiliations:** 1 Department of Biological Sciences and Center for Biotechnology and Interdisciplinary Studies, Rensselaer Polytechnic Institute, 110 8th Street, Troy, New York 12180, USA

## Abstract

Hydrostatic pressure is an important environmental variable that plays an essential role in biological adaptation for many extremophilic organisms (for example, piezophiles). Increase in hydrostatic pressure, much like increase in temperature, perturbs the thermodynamic equilibrium between native and unfolded states of proteins. Experimentally, it has been observed that increase in hydrostatic pressure can both increase and decrease protein stability. These observations suggest that volume changes upon protein unfolding can be both positive and negative. The molecular details of this difference in sign of volume changes have been puzzling the field for the past 50 years. Here we present a comprehensive thermodynamic model that provides in-depth analysis of the contribution of various molecular determinants to the volume changes upon protein unfolding. Comparison with experimental data shows that the model allows quantitative predictions of volume changes upon protein unfolding, thus paving the way to proteome-wide computational comparison of proteins from different extremophilic organisms.

## Abstract (web summary)

Proteins can be both stabilized and destabilized by pressure. Here the authors analyse the factors contributing to both negative and positive protein volume change upon denaturation, and shed light on the molecular determinants allowing proteins to be stable at high pressures.

Many extremophilic organisms, the so-called barophiles, evolved to live under high hydrostatic pressure^1^,^2^. These organisms generally populate the deep ocean floor where hydrostatic pressure can reach 1,100 atm^3^,^4^. Evolving to live under high hydrostatic pressure is not exclusive to single cell organisms. For example, the segmented microscopic animal tardigrade (‘water bear') can tolerate in their dormant state pressures up to 6,000 atm^5^. Pompeii worms (*Alvinella pompejana*) are species of polychaete worms that live at high pressures and temperatures near hydrothermal vents on the ocean floor^6^. Bacterial species have been isolated from 1,351 m into the Earth's crust where temperatures reach 102 °C and pressure is estimated to be in excess of 3,000 atm^7^,^8^. There are also reports of prokaryotic organisms at the bottom of oil well sediments and deep in the Arctic ice^9^.

What are the potential physicochemical implications for adaptation to high hydrostatic pressure? Biomacromolecules (proteins, DNA, RNA, lipid membrane) adopt unique three-dimensional structures (native or folded state) that are required for their biological function. The stability of these structures is very important for their function and thus biomacromolecules need to evolve to remain folded under the respective living conditions^4^,^10^,^11^,^12^. Increasing pressure, much like increasing temperature, perturbs the thermodynamic equilibrium between native folded state, *N*, and denatured unfolded state, *U*.


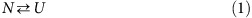


The response of the system to changes in pressure or temperature is governed by Le Chatelier's principle. Le Chatelier's principle is ‘*the tendency of a system to return to equilibrium by moving in the direction opposite to that caused by external perturbation*'^13^. For a two-state equilibrium between *N* and *U* states, the temperature response is described by a well-known van't Hoff equation.


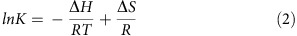


where Δ*H*=*H*_U_−*H*_N_ is the enthalpy change upon unfolding, *K* is the equilibrium constant and *R* is the universal gas constant. For a two-state equilibrium between *N* and *U* states, the pressure response is defined by a sign of the volume change upon unfolding, Δ*V*_Tot_,


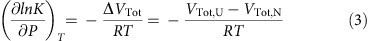


Combining equations (2) and (3) allows deriving the pressure–temperature phase diagram for protein stability^14^:


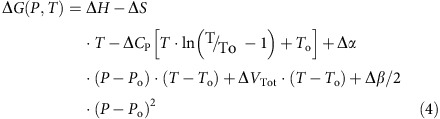


where Δ*H*, Δ*S* and Δ*V*_*Tot*_ are the unfolding enthalpy, entropy and volume changes at a reference temperature, *T*_o_, and reference pressure, *P*_o_, Δ*C*_p_ is the heat capacity change of unfolding. The reference temperature, *T*_o_, and pressure, *P*_o_, according to the biochemical convention are usually chosen to be 300 K and 0.1 MPa (=1 atm). The change in compressibility upon unfolding, Δ*β*, defined as Δ*β=*(γΔ*V*/γ*P*)_*T*_ is very small and often ignored^15^. The change in thermal expansivity upon unfolding, Δ*α*, defined as Δ*α=*(γΔ*V*/γ*T*)_*P*_ is known to be positive^16^. Therefore, Δ*V*_Tot_ is the key parameter in defining the effect of hydrostatic pressure on protein stability. According to Le Chatelier's principle, if Δ*V*_Tot_=*V*_Tot,U_−*V*_Tot,N_<0, the increase in pressure will shift the equilibrium from *N* to *U*, that is, will lead to a decrease in stability. Similarly, if Δ*V*_Tot_=*V*_Tot,U_−*V*_Tot,N_ >0, the increase in pressure will shift the equilibrium from *U* to *N*, that is, will lead to an increase in stability.

Here we will limit our discussion to only one type of biopolymers—proteins—but some of the issues can be pertinent to nucleic acids and to a lesser degree biological membranes. Experimental studies of the effects of hydrostatic pressure on protein stability have shown that most proteins unfold with an increase in pressure (negative Δ*V*_Tot_), although some are actually stabilized (positive Δ*V*_Tot_). The volume changes estimated from these experiments and also from direct measurements using pressure perturbation calorimetry are very small and range from −4% to +0.5%, relative to the total volume of the protein, that is, Δ*V*_Tot_*/V*_Tot,N_^16^,^17^,^18^,^19^. Such small in amplitude and variable in sign changes in Δ*V*_Tot_ have been puzzling the field for the past 50 years^20^,^21^,^22^. Since the first high-resolution structures of proteins have been solved, it has become evident that the native state of proteins contains a large number of voids (space inside protein that is not occupied by protein atoms) and cavities (voids large enough to accommodate a water molecule but may or may not be occupied by water)^23^. It has been shown that the volume fraction of the native protein that is occupied by voids ranges between 20 and 30%^23^,^24^,^25^. The presence of voids in the native state qualitatively explains the higher volume of the native state relative to the unfolded state. However, the magnitude of the negative volume changes due to the voids is much larger than the total negative volume changes observed experimentally and, moreover, cannot explain the experimentally observed positive volume changes. This led to a suggestion that possibly the volume changes due to the hydration of atoms inaccessible to the solvent in the native state and becoming exposed upon unfolding might lead to the positive contribution that offsets the negative void volume changes^26^,^27^. However, the transfer of model compounds from non-polar solvent into aqueous solution was also shown to be accompanied by negative volume changes^27^. This discrepancy between experimental volume changes and volume changes expected from protein structure and transfer studies has been termed the ‘Protein Volume Paradox'^27^,^28^. We performed detailed analysis of the two major assumptions ((1) all voids in the native state contribute to the total volume change upon unfolding and (2) hydration leads to a decrease in volume) that led to the formulation of this apparent paradox and showed that they are both oversimplified. We show that the volume changes upon protein unfolding can be calculated by explicitly modelling the volumes of the unfolded state ensemble and using a proper thermodynamic transfer model to account for the volume changes upon hydration.

## Results

### Definitions

**Figure 1:**
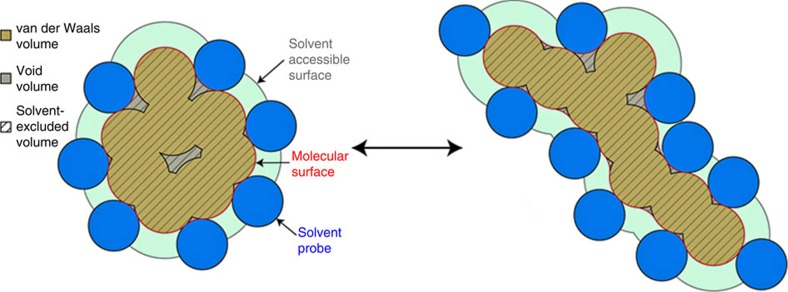
Pictorial definitions of volume changes upon protein unfolding. The volume enclosed by the molecular surface (red line) is the geometric or solvent-excluded volume (shaded area, *V*_SE_). Molecular surface is calculated by using solvent probe of 1.4 Å (blue spheres). The solvent-excluded volume consists of van der Waals volume (dark yellow area, *V*_vdW_), that is, the volume occupied by protein atoms, and void volume (grey area, *V*_Void_). Upon protein unfolding the molecular surface of the protein increases and some of the voids become solvent exposed.

The volume that a protein molecule occupies in solution is an additive quantity^23^ and can be separated into the geometric volume of the protein (*V*_SE_), and the volume changes in solvent due to the interactions with the protein surface (*V*_Hyd_). The geometric volume is the volume that is encompassed by the molecular surface as first defined by Richards^23^ and will be referred here as solvent-excluded volume (*V*_SE_). The *V*_SE_ of a protein comprises its van der Waals (*V*_vdW_) and void volume (*V*_Void_) (Fig. 1).


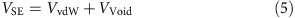


The van der Waals volume is the volume occupied by protein atoms, each given a specific van der Waals radius, while the void volume is the space inside the protein that is not occupied by protein atoms and is inaccessible to the solvent molecules modelled as spherical probes. The total volume of the protein in solution, *V*_Tot_, can be defined as the sum of two components:


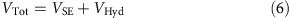


The volume changes upon protein unfolding are defined as the difference in total volumes of the unfolded, *V*_Tot,U_, and native, *V*_Tot,N_, states (Fig. 1).


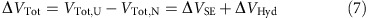


In the following section we will discuss the volumetric properties of the native and unfolded states and the contributions of the individual components, Δ*V*_SE_, and Δ*V*_Void_, to the total volume changes upon unfolding of proteins.

### Modelling the native state ensemble

**Figure 2:**
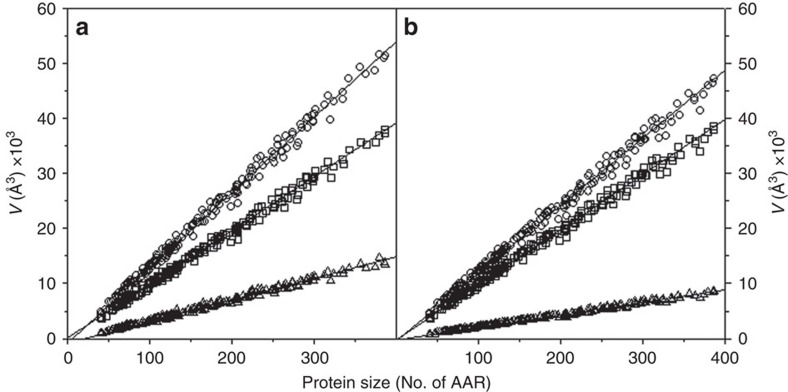
Breakdown of the contributions to the total geometric volume of protein. Contributions of van der Waals *V*_vdW_, squares) and void (*V*_Void_, triangles) volumes to the total geometric volume (*V*_SE_, circles) in the native (**a**) and unfolded (**b**) state ensembles as a function of protein size. Lines show linear regression fit. AAR, amino acid residue.

The properties of the native state are often modelled based on the X-ray and to a lesser degree NMR structures. However, the native state of a protein is not static, as portrayed by crystal structures, but dynamic. Thus, any property of the native state, such as volume, should be described by the properties of the ensemble. This allows the determination of not only the average property, but also the width of the distribution of volumes as reported by the s.d. Considering that molecular dynamics (MD) simulation is a well-established method to provide information about protein conformational fluctuations, we have used this method to model the fluctuations of native proteins^29^. As a test case for all calculations we have selected a non-redundant set of over 200 proteins of different size for which structures were solved with ultra-high (0.73–1.2 Å) resolution (see Supplementary Table 1 for the information on Protein Data Bank (PDB) structures used). For each protein, all-atom explicit solvent MD simulation was run starting from the X-ray coordinates as provided in the PDB file (see Methods for experimental details). The criterion for equilibration was the stable values of all-atom RMSD (root mean squared deviation) and stable values of solvent-excluded volume^30^. The average values of volume of structures from MD simulations were similar to the volumes obtained using the energy-minimized X-ray structure (Supplementary Fig. 1). The *V*_SE,N_ values for the native state ensembles scale linearly with protein size (Fig. 2a). The void volume also scales linearly with protein size (Fig. 2a) and the resulting packing density, defined as the ratio of van der Waals volume to solvent-excluded volume, varies between 0.7 and 0.8 (Supplementary Fig. 2) as was reported previously for other proteins^23^,^24^,^25^.

### Modelling of the unfolded state ensemble

To draw conclusions about the volumetric properties of the unfolded state, it is necessary to explicitly model the unfolded state ensemble. Experimental characterization of the structural properties of the unfolded state remains very challenging. Available indirect data suggest that the unfolded state can contain residual helical structure, hydrophobic clusters and native-like contacts^31^,^32^,^33^. Other studies showed that the dimensions of the unfolded state ensemble have properties characteristic of a random coil polymer or even statistical coil^34^,^35^,^36^. The random and/or statistical coil representation of the unfolded state can, arguably, be considered the most extended conformational ensemble.

**Figure 3:**
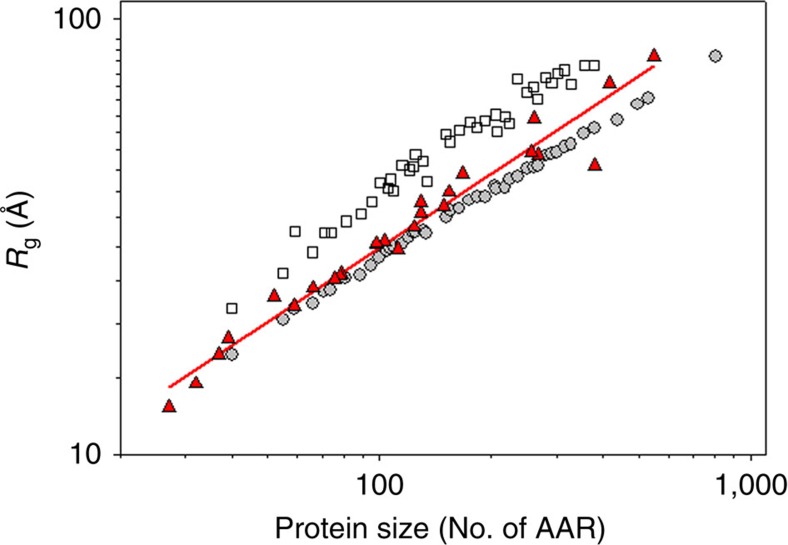
Radii of gyration (*R*_g_) of unfolded state ensembles generated using TraDES and SC compared with experimentally measured radii of gyration of unfolded proteins as a function of protein size. TraDES-generated unfolded state ensemble shows a dependence of radii of gyration (*R*_g_) on protein size similar to experimentally measured values^75^. Red triangles show the experimentally measured (using SAXS) values of *R*_g_ of proteins of various sizes. Open squares show the *R*_g_ values calculated using SC-generated ensemble, while grey circles show the values calculated using TraDES ensemble. For clarity only every fifth data point is shown. See also Supplementary Fig. 4 that shows the results for the FM-generated ensemble.

There are a number of ways to generate unfolded state ensembles^35^,^36^,^37^,^38^,^39^. It is well known that all-atom explicit solvent MD simulations produce overly compact or even highly helical structural ensembles that has been attributed to the artefacts of the current force-fields^40^. The empirical models or polymer-based models are free from such artefacts^39^. More importantly, the unfolded state ensembles, even those that differ by over 50% in the radius of gyration (*R*_g_), have very similar volumes (see Supplementary Fig. 3). This includes unfolded ensembles that incorporated significant fraction of helical structure. This underlines the fact noted by Fitzkee and Rose^35^ that *R*_g_ is a very coarse property to describe the unfolded state ensemble and is not a sufficient criterion to assess the lack of or presence of, for example, secondary structure in the unfolded state ensemble. This also suggests that the use of any of these ensembles will produce qualitatively similar volumetric results. Based on these initial observations and on the fact that three computational models, statistical coil model of the unfolded state (SC)^36^, trajectory directed ensemble sampling (TraDES)^37^ and flexible-meccano (FM)^38^, have been benchmarked against NMR-derived parameters such as chemical shifts and J-coupling constant^36^,^41^,^42^, we generated 1,000 unfolded conformations for each protein in our data set (see Supplementary Table 1 for the list of proteins). As expected, all ensembles show a lack of specific long-range contacts or secondary structure (see Supplementary Fig. 3). Furthermore, *R*_g_ compares well with the experimentally determined power-law dependence of *R*_g_ on protein size (that is, number of amino acid residues, see Fig. 3). The SC ensemble produces somewhat higher values of *R*_g_^36^,^39^, while TraDES and FM produce smaller values of *R*_g_ and show remarkable agreement with the experimental values. There is ample evidence that pressure and chemical unfolding of proteins results in similar volume change^43^. Thus the unfolded state ensembles generated this way are consistent with the only currently available experimental data, that is, power law scaling of *R*_g_ for polymers in a good solvent. Although TraDES and FM show the closest agreement with experimental *R*_g_ values (Fig. 3 and Supplementary Fig. 4), due to limitation in the maximum sequence length in FM, TraDES was selected for all detailed calculations as a model of the unfolded state ensemble.

The *V*_SE,U_ values for the unfolded state ensemble also scale linearly with protein size but, as expected, the dependence is less steep than *V*_SE,N_ (see Fig. 2a,b). Importantly, the unfolded state ensemble also contains a significant amount of void volume that also scales linearly with the protein size (Fig. 2b). The packing density of the unfolded state ensemble (see Supplementary Fig. 2), defined as a ratio of van der Waals volume to solvent-excluded volume, is very uniform, 0.824±0.005 (max=0.841; min=0.815).

### Calculating Δ*V*_SE_

The *V*_SE_ of a protein describes the geometric or structural contribution to protein volume. The changes in this volume upon protein unfolding then can be calculated by subtracting the *V*_SE_ values of *U* and *N* state ensembles:


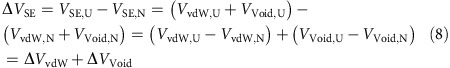


**Figure 4:**
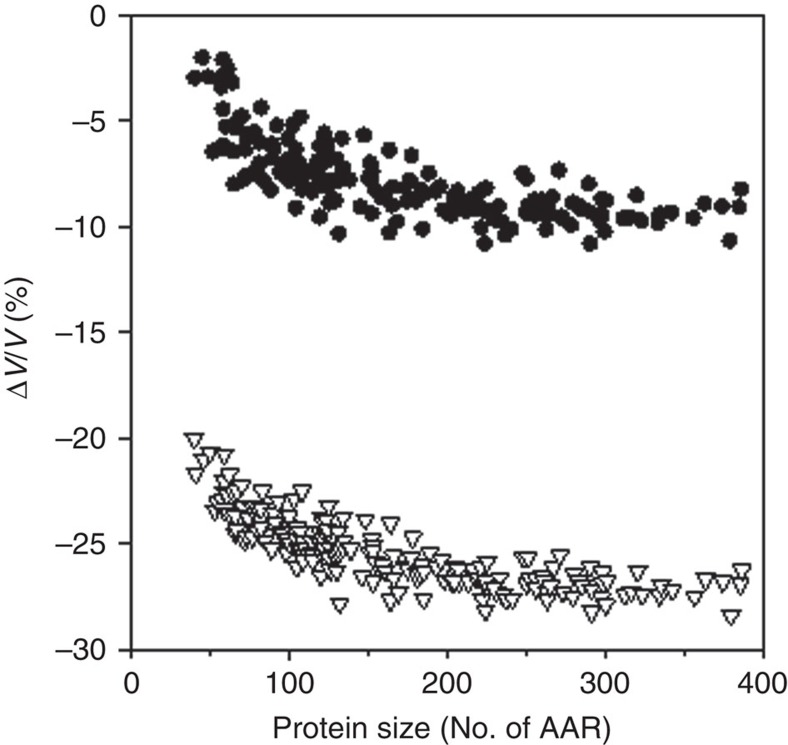
Void volume changes upon unfolding as a function of protein size. Comparison of the void volume changes expected by considering that all void volume of the native protein contributes to the Δ*V*_Void_*=*−*V*_Void,N_, upon unfolding (triangles) with the volume changes that explicitly take into account the void volume of the unfolded state ensemble Δ*V*_Void_*=V*_Void,U_−*V*_Void,N_ (circles).

By this definition, the Δ*V*_SE_ has two contributions, one from changes in the van der Waals volume, Δ*V*_vdW_, and another from the changes in void volume, Δ*V*_Void_. The van der Waals volume of the native state is always slightly lower than the van der Waals volume of the unfolded state (Fig. 4). This lower *V*_vdW_ of the native state is mainly associated with the extensive intramolecular hydrogen bonding in the native state^30^,^44^. Indeed, there is direct correlation between the difference in the average number of hydrogen bonds in the native and unfolded ensembles and the Δ*V*_vdW_ (Supplementary Fig. 5). However, the Δ*V*_vdW_ will not contribute to the total volume changes upon protein unfolding, Δ*V*_Tot_, because of the hydrogen bonding with the solvent in the unfolded state that is of a similar magnitude and part of the hydration volume, Δ*V*_Hyd_.

As expected, the difference in void volumes between unfolded and native state Δ*V*_Void_ is negative. When the polymer properties of the unfolded state ensemble are explicitly taken into account, Δ*V*_Void_*=V*_Void,U_−*V*_Void,N_, the change in void volume upon protein unfolding is much smaller (−7±2%) than the −20 to −30% previously defined by the elimination of all void volume of the native state (Δ*V*_Void_*=*−*V*_Void,N_)(Fig. 4). This suggests that the first postulate in the Protein Volume Paradox grossly overestimates the contribution of void volume to the total volume changes upon protein unfolding.

### Calculating volume of hydration Δ*V*_Hyd_

**Figure 5:**
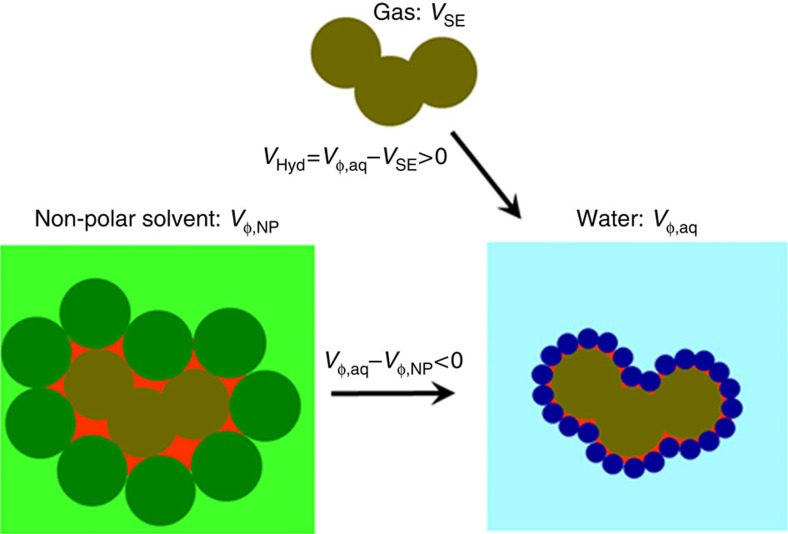
Pictorial definition of hydration volume. Volume of solute in non-polar phase (*V*_φ,NP_) includes volume that a solute occupies in non-polar solvent (red area). The difference between the volume in the gas phase (*V*_SE_) and the partial volume of solute in water (*V*_φ,aq_) accounts only for the volume changes due to the interactions with water. Thus, the hydration volume can be defined as *V*_Hyd_*=V*_φ,aq_**−***V*_SE_.

**Figure 6:**
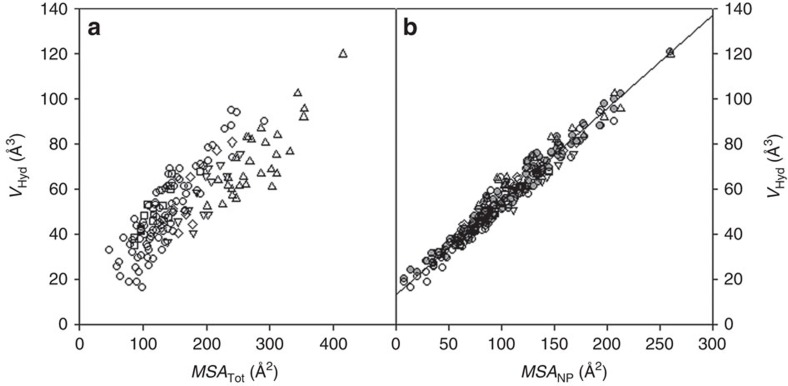
Hydration volume of model compounds. The dependence of hydration volume, *V*_Hyd_, of model compounds at 25 °C on the total (*MSA*_Tot_, (**a**)) or non-polar (*MSA*_NP_, (**b**)) surface area shows that non-polar groups make a major contribution. Aromatic model compounds (▪); non-aromatic model compounds (circle); oligopeptides (*n*=3–5) (triangle); *N*-acetyl amides of amino acids (upside-down triangle); and *N*-acetyl amino acids (diamond). In **b**, the scatter in the grey circles calculated with equation (9) matches the scatter in *V*_Hyd_ data and the line shows linear regression of *V*_Hyd_ versus *MSA*_NP_.

When a protein unfolds, groups buried in the native state become exposed and will interact with water. These interactions can possibly lead to volume changes and thus must be accounted for when considering volume changes upon protein unfolding. Historically, this unfolding reaction was modelled as a transfer from non-polar solvent to water^20^,^21^,^27^. It has been shown that volume change upon such transfer, usually attributed to the volume changes upon hydration, is always negative, that is, volume of a solute in aqueous solution is always smaller than that in non-polar solvent^20^,^21^,^27^. However, transfer from non-polar solvent to aqueous solution has been proven to be inadequate to model thermodynamics of hydration (see for example, refs 45, 46). In the case of volume changes, the larger size of non-polar solvent than that of water leads to an overestimation of the volume that a solute occupies in the non-polar phase^47^ (see Fig. 5). It has been well established that transfer from gas phase into aqueous solution is a more appropriate way to model thermodynamics of hydration^46^,^48^,^49^,^50^. This is also the case for modelling the volume changes upon hydration (Fig. 5). To understand the volume changes upon hydration in more detail, we analysed the experimentally measured volume change upon transfer of over 150 different model solutes (aromatic and non-aromatic compounds, oligopeptides and *N*-acetyl amides of amino acids) from gas phase into aqueous solution at 25 °C^51^. The hydration volume upon transfer from gas phase into water, *V*_Hyd_, is equal to the experimentally measured partial volume of a solute in water minus the geometric volume of this solute (that is, *V*_SE_) (Fig. 5). This volume will, in a first approximation, depend on the number of water molecules that can directly interact with the solute and thus is proportional to the molecular surface area of the solute molecule. Figure 6a shows the dependence of the hydration volume, *V*_Hyd_, on the total molecular surface area (MSA) of various solutes, *MSA*_Tot_. Two major observations can be made from the plot shown in Fig. 6a. First, the hydration volume is always positive, that is, transfer of any (polar or non-polar) solute into aqueous solution leads to an increase in volume. Second, the increase in the molecular surface area leads to an increase in the corresponding hydration volume. However, there is a large spread in this trend (Fig. 6a). Interestingly, the correlation becomes much more pronounced (*R*^2^=0.94) if we plot only non-polar MSA (*MSA*_NP_) as a function of *V*_Hyd_ (see Fig. 6b). This suggests that the solvent interactions with non-polar surfaces make up a major contribution to the volume changes upon hydration. This observation is in line with some previous reports^52^; however, present analysis has been done on a much larger and more diverse data set. Importantly, not all variance in *V*_Hyd_ can be explained by *MSA*_NP_. Therefore, we performed a fit to both non-polar, *MSA*_NP_, and polar, *MSA*_Pol_, surface areas


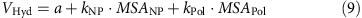


where *k*_NP_ and *k*_Pol_ are the contributions of a given type of MSA to the hydration volume for non-polar and polar surfaces, respectively. The fit to equation (9) provides a much better description (*R*^2^=0.97) of the variance in *V*_Hyd_ (see Fig. 6b) than just a one parameter fit using only non-polar MSA. The *k*_NP_ coefficient is larger (0.38±0.2 Å) than *k*_Pol_ (0.03±0.03 Å). The small value for the polar coefficient can be easily rationalized as hydrogen bonding of water with polar groups, while the large value of the *k*_NP_ coefficient suggests that water molecules move away from non-polar groups due to the hydrophobic effect^30^,^53^,^54^,^55^.

**Figure 7:**
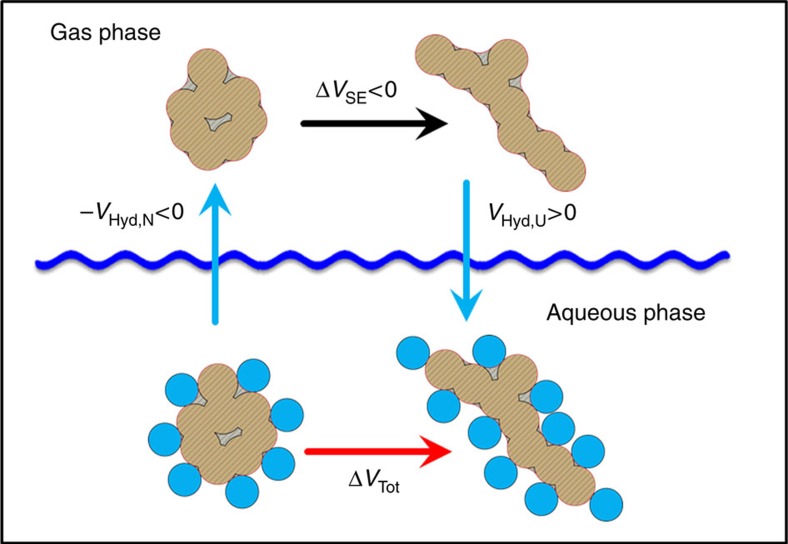
Thermodynamic cycle for separating the contributions of hydration of native (V_Hyd,N_) and unfolded (V_Hyd,U_) state ensembles from the contribution of geometric volume changes (ΔV_SE_) to the total changes in volume (ΔV_Tot_) upon protein unfolding in aqueous solution. The sum of all three steps is equal to the volume of unfolding of the protein in aqueous solution, Δ*V*_Tot_, as defined by equation (11). It must emphasized that this process is valid because volume as a thermodynamic parameter is a state function and there are no conformational changes in the native or unfolded state ensembles upon transfer to and from the gas phase.

Having empirically established the contribution of polar and non-polar groups to the volume of hydration and, assuming that such proportionality can be extrapolated to much larger surfaces, we can calculate the volume changes due to changes in hydration upon protein unfolding. Volume as a thermodynamic quantity is an additive state function that allows for the use of the thermodynamic cycle shown in Fig. 7. In this hypothetical thermodynamic transfer cycle, a native state ensemble is first transferred into the gas phase. This step essentially accounts for the volume change upon dehydration of the native state ensemble. The volume changes are calculated as:

**Step 1** 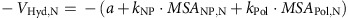


where the minus sign signifies that the transfer is from aqueous phase into gas phase (the opposite of hydration, that is, dehydration).

The second step is unfolding of a protein in the gas phase that corresponds to the changes in the geometric volume:

**Step 2** 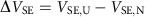


Finally, the unfolded state ensemble is transferred back to aqueous solution. This step corresponds to volume change upon hydration of the unfolded state ensemble and is calculated as

**Step 3** 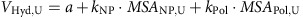


The sum of steps 1 and 3 reflects the change in hydration upon unfolding. Assuming that the proportionality coefficients *k*_NP_ and *k*_Pol_, derived from model compounds (that is, equation (9)), can be extrapolated to much larger surfaces, the changes in hydration volume upon unfolding can be calculated as:


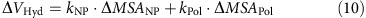


where Δ*MSA*_NP_*=MSA*_NP,U_−*MSA*_NP,N_ and Δ*MSA*_Pol_*=MSA*_Pol,U_−*MSA*_Pol,N_ are the differences in the non-polar and polar surface areas of the unfolded and native states, respectively. These can be calculated using the native and unfolded state ensembles modelled as described in previous sections. The resulting Δ*V*_Hyd_ is always positive due to two factors: (1) the MSA of the unfolded state is larger than the MSA of the native state and (2) both *k*_NP_ and *k*_Pol_ coefficients are positive. This suggests that the second postulate in the Protein Volume Paradox misrepresents the contribution of hydration volume (that is actually positive and not negative) to the total volume changes upon protein unfolding.

### Comparison of computed and experimental values for Δ*V*_Tot_

**Figure 8:**
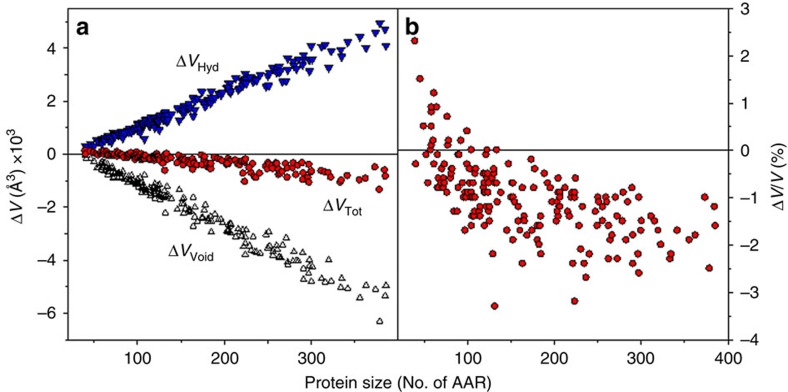
The dependence of the total volume changes upon unfolding (Δ*V*_Tot_ calculated using equation (11)) on protein size. (**a**) Contributions of void (Δ*V*_Void_, triangles) and hydration (Δ*V*_Hyd_, upside-down triangles) volume changes to the total volume (Δ*V*_Tot_, circles) changes upon unfolding as a function of protein size. (**b**) Fractional changes in the total volume Δ*V*_Tot_/*V*_Tot,N_ as a function of protein size.

Results presented in the previous sections allow us to calculate the expected total changes in volume upon protein unfolding as:


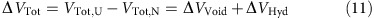


and analyse the relative contributions of each component. Figure 8a shows the protein size dependence of the calculated changes in the Δ*V*_Tot_ and analyses the contributions from the changes in void, Δ*V*_Void_, and hydration, Δ*V*_Hyd_, volumes. The contribution of void volume to the total volume upon protein unfolding is relatively large and negative. It originates from the fact that there is larger void volume in the native state than in the unfolded state ensemble, represented here using the maximally unfolded polymer-based model (see also Figs 3 and 4). The volume changes due to hydration are also positive and comparable in absolute values to the corresponding absolute values of Δ*V*_Void_. This leads to rather small relative volume changes upon unfolding (Fig. 8b). Importantly, the balance between these two factors can produce both positive and negative changes in the total volume upon protein unfolding. This is in excellent qualitative agreement with the general experimental observations that proteins can be both stabilized (positive Δ*V*_Tot_) and destabilized (negative Δ*V*_Tot_) by hydrostatic pressure^16^,^17^,^18^,^19^,^56^,^57^.

**Figure 9:**
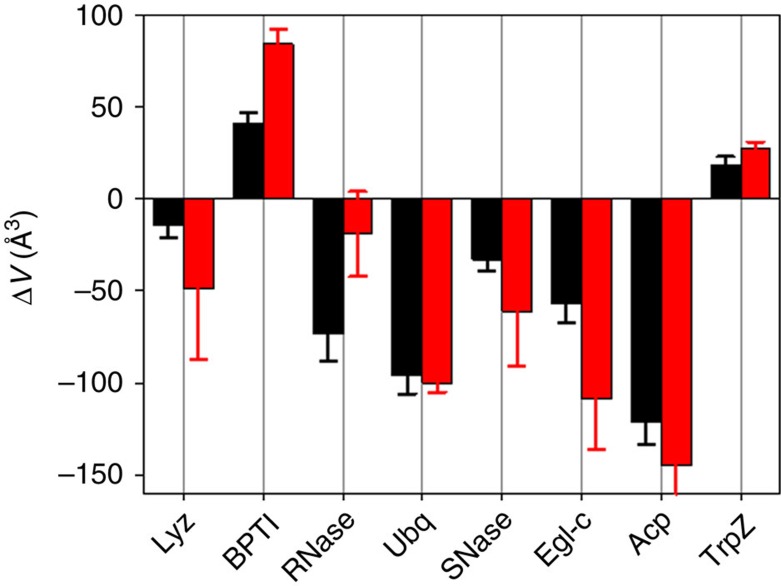
Direct comparison of experimentally measured (black bars) and calculated using equation (11) (red bars) volume changes upon unfolding of eight globular proteins. Lyz, hen egg white lysozyme PBD:4LZT; BPTI, bovine pancreatic trypsin inhibitor PBD:6PTI; RNAse, bovine pancreatic ribonuclease A PDB:7RSA; Ubq, human ubiquitin PDB:1UBQ; SNase, ΔPHS variant of staphylococcal nuclease PDB:3BDC; Egl-c, leech eglin c PDB:1EGL; Acp, human acylphosphatase PDB:2ACY; TrpZ, Tryptophan Zipper PDB:1LE3. Experimental data (25 °C SNase; 40 °C RNase; 50 °C Acp, Egl and Lyz; 90 °C BPTI; and 80 °C TrpZ) are taken from refs 16, 57, 58. It is important to note that volume changes are temperature dependent^19^ that can also contribute to the observed differences between experimental and calculated values. Error bars show s.d. of averaging the experimental data over measured temperature range or of the multiple (*n*=3–8) repeats of MD runs for the native state (see Methods section for details).

To test the reliability and accuracy of the presented formalism we have compared the calculated (using equation (11)) and experimentally measured values of volume change upon unfolding for eight proteins: lysozyme, pancreatic trypsin inhibitor, ribonuclease A, ubiquitin, acylphosphatase, eglin c, tryptophan zipper and staphylococcal nuclease Δ+PHS variant^16^,^57^,^58^. Figure 9 shows that predicted values are in very good agreement with experimental values. In particular, the predicted values recapitulate not only the sign of the volume changes but also the magnitude. Thus, the formalism to calculate the volume changes upon protein unfolding presented here appears to provide near quantitative prediction of the expected volume changes. Furthermore, our results present a quantitative molecular picture of how hydrostatic pressure modulates the conformational equilibrium of proteins. Finally, the structure-based prediction of volume changes upon protein unfolding can now be applied to a proteome-wide comparison of proteins from organisms living under ambient pressure with those from organisms that evolved to live under extreme pressures. The expectation is that the proteins from barophilic organisms could have evolved to have volume changes upon unfolding that were either less negative or even positive relative to those of the mesophilic homologues, by decreasing the void volume in the native state and/or increasing the fraction of buried non-polar residues.

## Methods

### Sampling of native and unfolded ensembles

A non-redundant set of proteins with X-ray structures solved to ultra-high resolution (0.73–1.2 Å resolution, see Supplementary Table 1) was selected for modelling.

Native state all-atom explicit solvent MD simulations were carried out in GROMACS 4.6.3 (ref. 59) using the CHARMM27 force field and TIP3P water model^60^. The native crystal structure was solvated in a dodecahedron box, with dimensions such that all protein atoms are at least 10 Å deep in the box, and neutralized with 0.1 M excess NaCl, followed by energy minimization for 1,000 steps. All simulations underwent 200 ps of constant volume equilibration, 100 ps of constant pressure equilibration and 50 ns of production simulation at 300 K and 1 bar. We used the Parrinello–Rahman^61^ pressure control with a 2 ps relaxation time and a compressibility of 4.6 × 10^−5^ atm^−1^, and v-scale temperature coupling^59^. LINCS^62^ and SETTLE^63^ algorithms were used to constraint high-frequency bond vibrations that allowed the use of a 2 ps integration step. The electrostatic interactions were modelled by the smooth particle mesh Ewald method^64^, using a 75 × 75 × 75 grid, with fourth-order charge interpolation and a real space cutoff of 1.0 nm. A native ensemble of 50 structures was extracted from the production trajectory (1 structure per ns). Equilibration criteria were stable all-atom r.m.s.d. with respect to the crystal structure (< 2 Å) and stable values (drift 0.5%) of solvent-excluded volume throughout the production trajectory. The low-frequency motions are expected to introduce only small corrections to the solvent-excluded volume, though further study would be warranted for proteins that undergo large-scale conformational fluctuations.

Sampling of the unfolded state ensembles was carried out using three generators: TraDES^37^, SC^36^ and FM^38^. Each unfolded state ensemble consisted of 1,000 structures. TraDES ensembles were generated with the all-coil sampling flag (-c T) to remove all secondary structure propensity. Coil and FM ensembles were generated using default settings. SC and FM generate only the protein backbone, and hence Scwrl4 (ref. 65) was used to add side-chains. Before volume calculations, the structures generated by TraDES, SC or FM were energy minimized in implicit solvent, using the Generalized Born surface area (GBSA) model with protein and solvent dielectric constants of 80 in GROMACS 4.6.3. This step also explicitly incorporates all hydrogen atoms. The disulfide bridges for three individual proteins (BPTI, RNase and Lyz) modelled into the TraDES unfolded state ensemble by performing restrained all-atom MD simulations using the following protocol in GROMACS 4.6.3 with TIP3P water and CHARMM27 force field. First, the structures were energy minimized for 800 steps before enabling distance restraints. Distance restraints were gradually shortened in four 5 ps steps, where distance restraints were 3, 1, 0.5 and 0.2 nm, respectively. Upon reaching 0.2–0.3 nm distance between sulfur atoms, the structure was energy minimized in the CHARMM27 force field for 1,000 steps in implicit solvent, using the steepest descent algorithm.

### Model compounds

The experimentally measured at 25 °C partial molar volumes in aqueous solution, *V*_φ,aq_, of 150 model compounds relevant to proteins (alkanes, aromatic compounds, alcohols, diols, amines, amides, diamines, diamides, dicarboxylic acids, hydroxyamides, hydroxy acids, ketones, polyethylene glycols and ureas) were taken from refs 51, 66. For each of these model compound PDB structures were generated using the CORINA webserver^67^. In addition, we included into our analysis more complex model compounds such as oligopeptides (3-5 residues), *N*-acetyl amino acid amides and *N*-acetyl amino acids that represent protein components, for which the experimentally measured partial molar volumes in aqueous solution, *V*_φ,aq_, are also reported^15^,^68^,^69^. For these compounds, structures were generated using the same protocol as for the native proteins (see above). The hydration volume is calculated as a difference between partial molar volume of a compound in aqueous solution, *V*_φ,aq_, and corresponding solvent-excluded volume, *V*_SE_, (see also Fig. 5). MSMS software package^70^ was used to analytically calculate MSA per atom for all structures of model compounds. Each structure had its MSA broken down into carbon, nitrogen, oxygen and sulfur surface areas. Hydrogen surface areas were combined with the surface area of their parent heavy atom. Carbon and sulfur molecular surface areas were combined into non-polar MSA (*MSA*_NP_). Nitrogen and oxygen molecular surface areas were combined into polar MSA (*MSA*_Pol_).

### ProteinVolume calculations

The ProteinVolume software package has been described by us previously^71^. Briefly, it uses a flood-fill algorithm to first calculate the molecular surface based on the coordinates, and then fill the enclosed volume with 0.02 Å probes. The sum of all volume probes is calculated and reported as the solvent-excluded protein volume (*V*_SE_). Van der Waals volume (*V*_vdW_) is also calculated during the same step as the solvent-excluded volume calculation procedure, but with an additional check of whether the volume probe is within the van der Waals radius of a protein atom. A probe that lies on top of a van der Waals boundary is stochastically accepted with the acceptance probability based on its magnitude of overlap with the atom. This increases the accuracy of the van der Waals volume calculation and reduces the volume underestimation of numerical integration methods. The sum of all van der Waals volume probes is calculated and reported as van der Waals protein volume (*V*_vdW_). Void volume, *V*_Void_, is calculated as the difference between the solvent-excluded volume and the van der Waals volume. ProteinVolume uses all-atom Bondi radii set^72^. United atom radii are slightly larger to represent the additional radius of bonded hydrogen atoms averaged across the entire atom surface and this leads to an overestimation of van der Waals volume. Volume changes upon ionization (electrostriction) vary between different protein groups (see Supplementary Table 2). Importantly, most charged groups are on the surface of native proteins, and thus at most 1–2 protons will be released or absorbed upon protein unfolding. This might have an effect for smaller proteins <100 residues, but small proteins rarely have buried charged groups^73^. Continuum electrostatics calculations using h++ server^74^ of net charge in the native and unfolded ensembles for eight proteins that were quantitatively compared with the experimental ΔV values (for example, Fig. 9) show that there is no change in the net charge upon unfolding of these proteins (see Supplementary Fig. 6).

### Data availability

The data that support the findings of this study are available from the corresponding author on reasonable request.

## Additional information

**How to cite this article:** Chen, C. R. & Makhatadze, G. I. Molecular determinant of the effects of hydrostatic pressure on protein folding stability. *Nat. Commun.* **8,** 14561 doi: 10.1038/ncomms14561 (2017).

**Publisher's note:** Springer Nature remains neutral with regard to jurisdictional claims in published maps and institutional affiliations.

## Supplementary Material

Supplementary Information Supplementary Figures, Supplementary Tables and Supplementary References
