# Supplementary material for: Molecular determinant of the effects of hydrostatic pressure on protein folding stability
Source: Nat Commun. 2017 Feb 7;8:14561. doi: 10.1038/ncomms14561 (PMC5309723; doi:10.1038/ncomms14561)
Supplement: Supplementary Information — Supplementary Figures, Supplementary Tables and Supplementary References [file ncomms14561-s1.pdf]

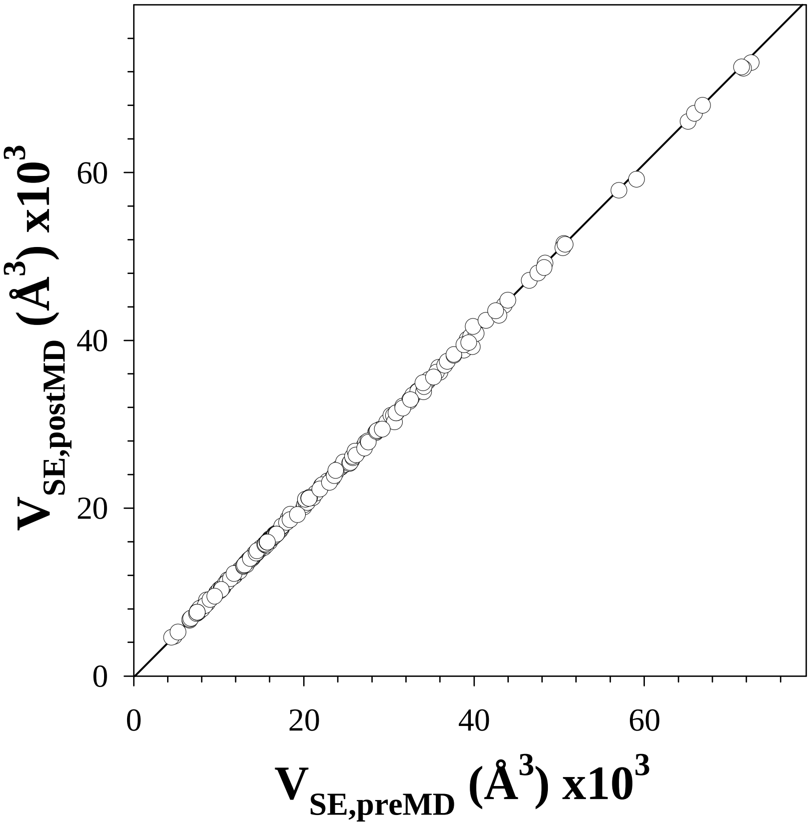

Supplementary Figure 1. Comparison of the solvent-excluded volumes of proteins in the native state based on the x-ray structure ($V_{\mathrm{SE,preMD}}$) with the solvent-excluded volumes calculated using native state ensemble obtained using MD simulations ($V_{\mathrm{SE,postMD}}$). The standard deviations of $V_{\mathrm{SE,postMD}}$ values are smaller than the symbol size.

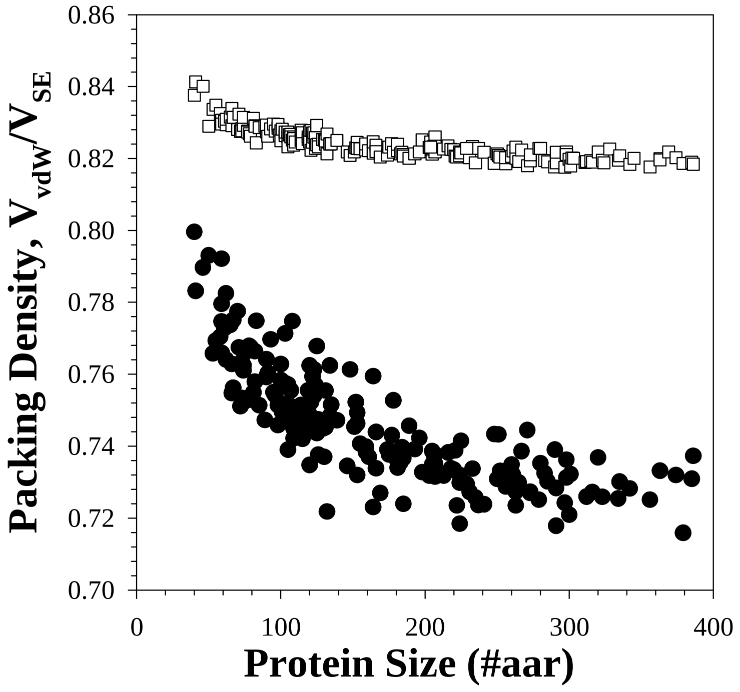

Supplementary Figure 2. Comparison of packing densities of the native (circles) and unfolded (squares) state ensembles. Packing density is defined as the ratio of the van der Waals volume to solvent-excluded volume.

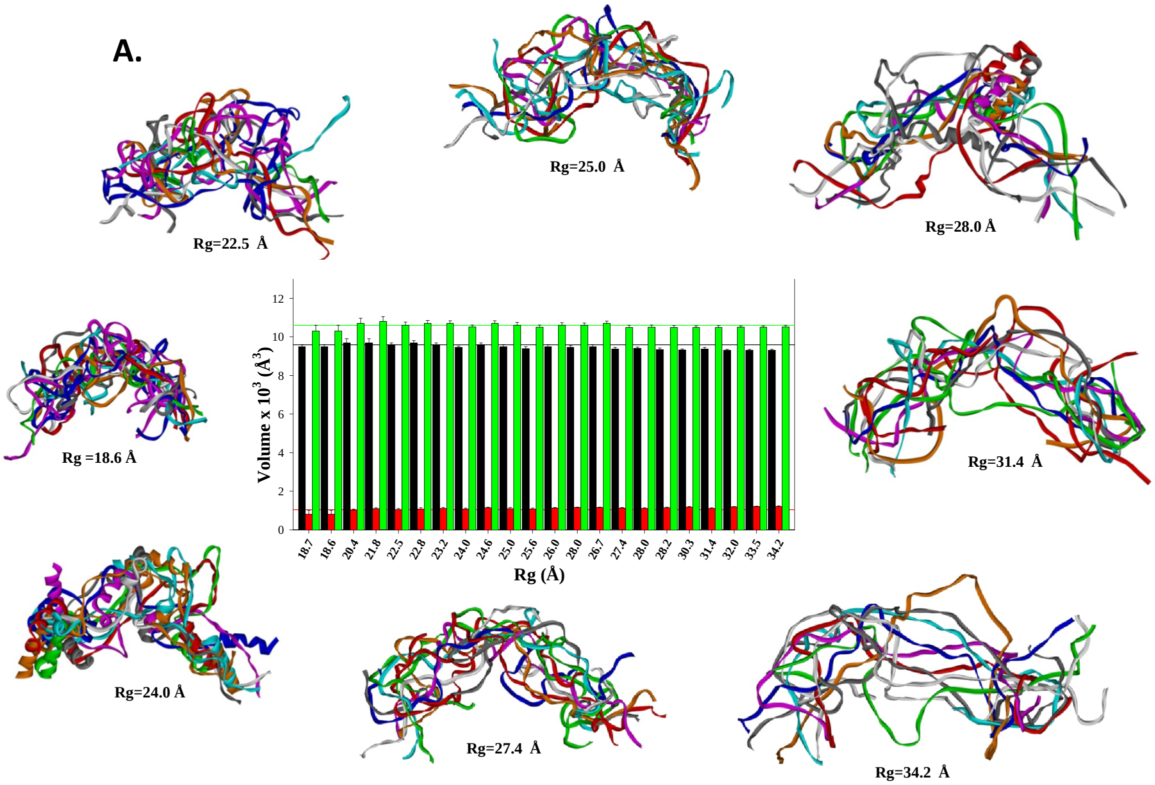
A.
Rg=25.0 Å
Rg=22.5 Å
Rg=28.0 Å
Rg =18.6 Å
Rg=31.4 Å
Volume x 10^3 (Å^3)
Rg (Å)
18.7
18.6
20.4
21.8
22.5
22.8
23.2
24.0
24.6
25.0
25.6
26.0
28.0
26.7
27.4
28.0
28.2
30.3
31.4
32.0
33.5
34.2
Rg=24.0 Å
Rg=27.4 Å
Rg=34.2 Å

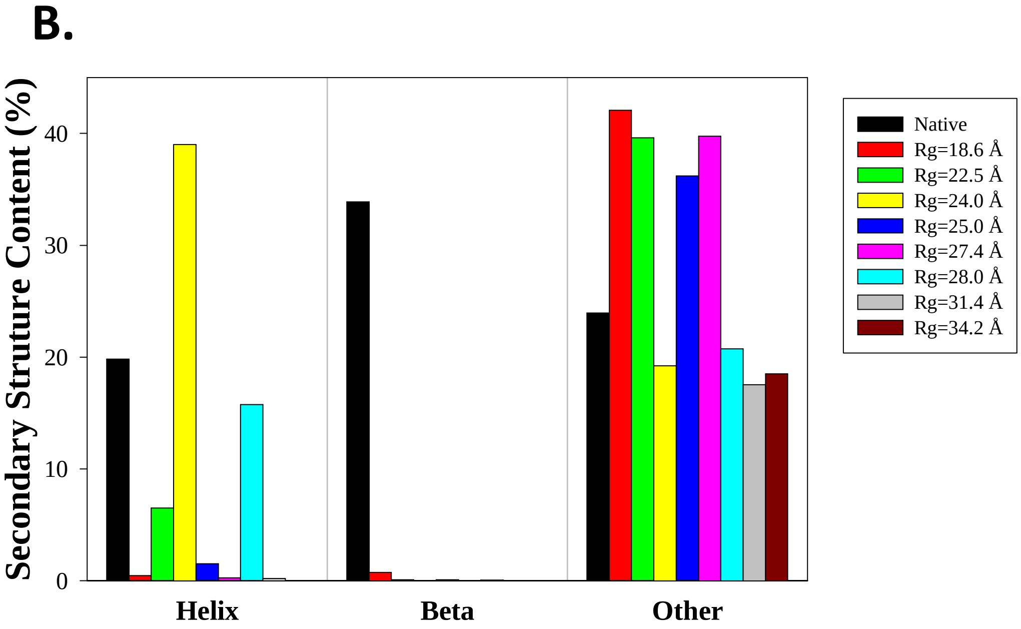
B.
Secondary Struture Content (%)
Helix
Beta
Other
Native
Rg=18.6 Å
Rg=22.5 Å
Rg=24.0 Å
Rg=25.0 Å
Rg=27.4 Å
Rg=28.0 Å
Rg=31.4 Å
Rg=34.2 Å

C.

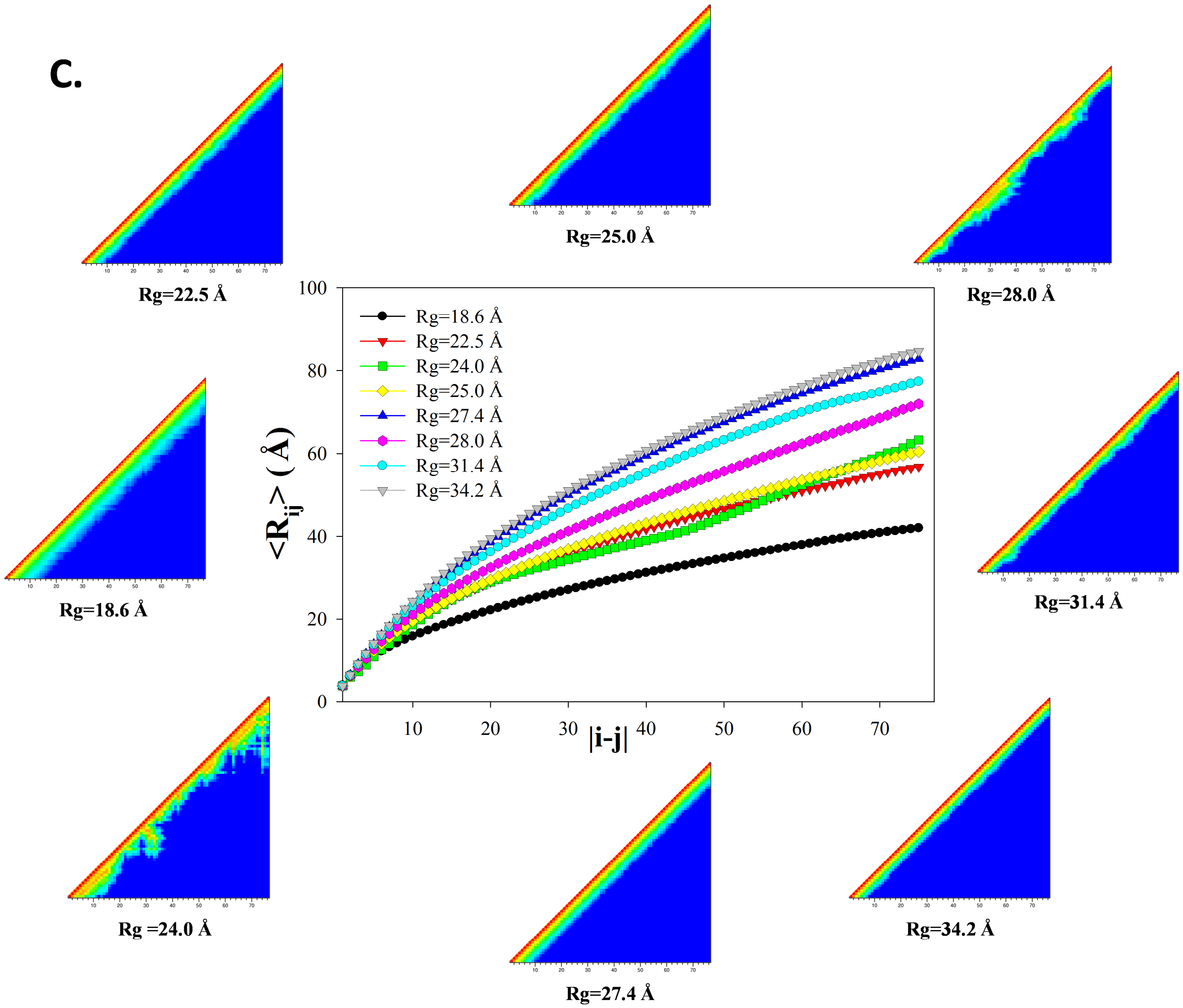

Supplementary Figure 3. Characterization of various unfolded state ensembles generated using TraDES [1] (Rg=22.5 Å), flexible-meccano [2] (FM, Rg=25.0 Å), Statistical Coil [3] (SC, Rg=31.4 Å), Fitzkee & Rose[4] approach (Rg=24.0), Fitzkee & Rose-like approach (Rg=28.0 Å), or CAMPARI [5, 6] (Rg=18.6, 27.4 and 34.2 Å). The listed CAMPARI simulations at R_g=18.6 Å and 34.2 Å nm were generated by sampling the Theta solvent and EV (excluded volume) ensembles, respectively. The Theta solvent ensemble was generated by turning off attractive and repulsive LJ forces and randomly sampling backbone dihedral angles from grid files for each respective amino acid type. The EV ensemble was generated by setting attractive LJ force to 0 and repulsive LJ force to 1. The simulation at R_g=27.4 Å was generated by setting the attractive LJ force to 0 and repulsive LJ force to 0.001. The Fitzkee/Rose-like simulations were carried out by generating an all-atom structure-based model from the crystal structure of using SMOGv2[7, 8]. All pairwise interactions were removed so the Hamiltonian has only parameters for bonds, angles, and dihedral angles remaining. Residues with secondary structure were identified in the native

crystal structure and had their respective dihedral angles fixed in the structure-based model. The simulation was then run at 120K to generate an unfolded state that still maintained its native secondary structure. Data, shown for illustrative purposes, was computed from the conformational ensembles of ubiquitin (PDB:1UBQ). **Panel A** shows ribbon traces of 10 representative structures from each structural ensemble with the corresponding ensemble average Rg. Central insert shows a comparison of the volumes for these ensembles. V_{SE} (black bars) V_{Hyd} (red bars) and V_{Tot} (green bars). In addition, volumes of other ensembles, generated using CAMPARI, with intermittent Rg are also shown. **Panel B**. Comparison of secondary structure content in the unfolded state ensembles. Secondary structure content was calculated using DSSP 2.2.1 [9]. Helical structure is the sum of 3-, 4-, and 5-turn helices, sheet structure is the sum of β-bridges and β-sheets, and other includes turn and bend. **Panel C**. Comparison of contact maps of the unfolded ensembles calculated as averaged distance matrices consisting of the smallest distance between residue pairs generated using the GROMACS utility g_mdmat. Central insert shown fractal dimensions of the unfolded state ensembles calculated as the average distance $\langle R_{ij} \rangle$ between Cα atoms of residues i and j as a function of sequence separation $|i-j|$ [10].

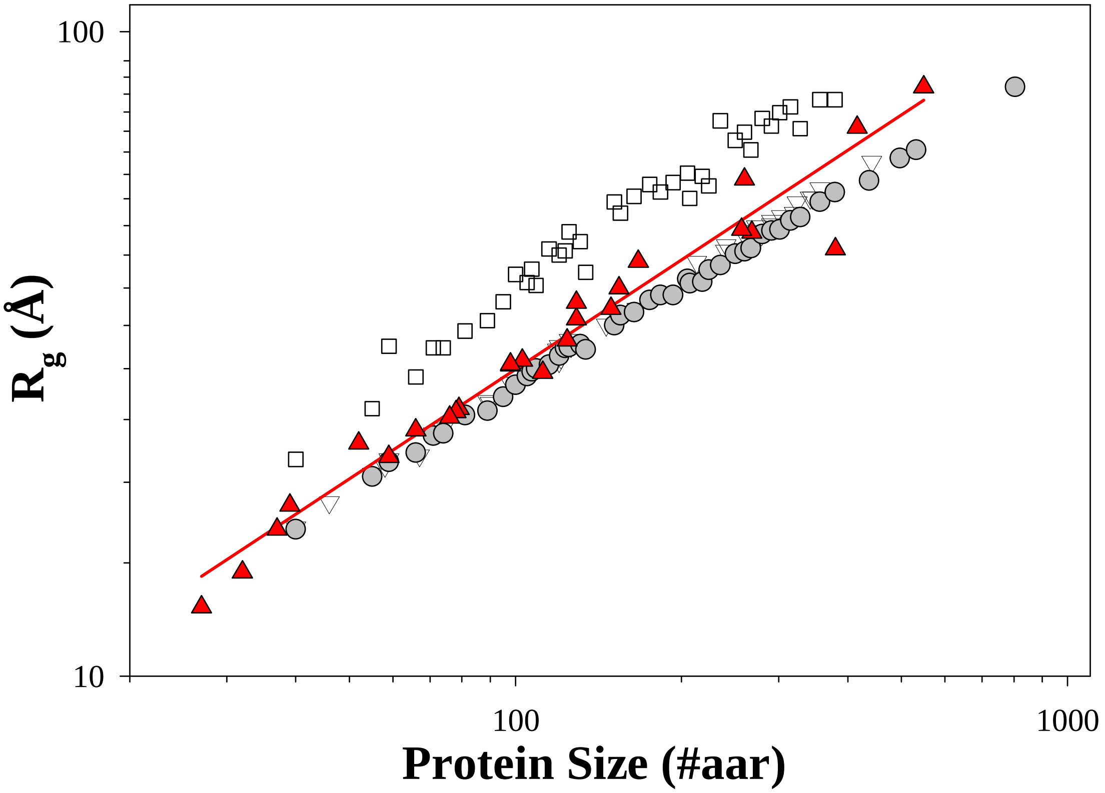

Supplementary Figure 4. TraDES and FM generated unfolded state ensembles show a dependence of radius of gyration (R_g) on protein size similar to experimentally measured values [11]. Red triangles show the experimentally measured (using SAXS) values of R_g for proteins of various sizes. Open squares show the R_g values calculated for the SC-generated ensemble, while gray circles show the values calculated for the TraDES ensemble, and open triangles show the results for the FM-generated ensemble. For clarity only every fifth data point is shown.

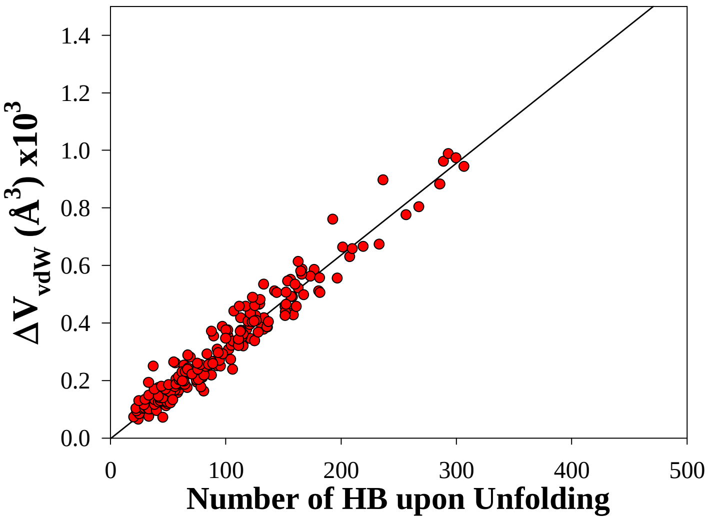

Supplementary Figure 5. Difference in the van der Waals volumes of native and unfolded state ensembles (ΔV_{vdW}) is due to the larger number of hydrogen bonds in the native relative to the unfolded state ensemble. Dependence of ΔV_{vdW} on the average change in the number of hydrogen bonds upon unfolding is linear with a Pearson correlation coefficient of 0.96. The average number of hydrogen bonds for each ensemble was calculated using DSSP 2.2.1 [9].

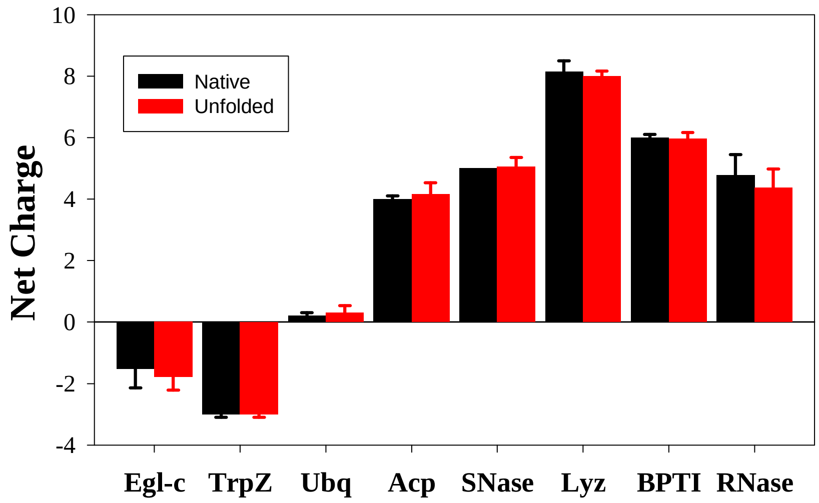

Supplementary Figure 6. Comparison of the net charge of the native and unfolded state ensembles calculated using h++ server [12, 13]. Error bars are the standard deviations of the mean.

References

1. Feldman H, Hogue C. Probabilistic sampling of protein conformations: New hope for brute force? *Proteins* **23**, 8-23 (2002).

2. Ozenne V*, et al.* Flexible-meccano: a tool for the generation of explicit ensemble descriptions of intrinsically disordered proteins and their associated experimental observables. *Bioinformatics* **28**, 1463-1470 (2012).

3. Jha AK, Colubri A, Freed KF, Sosnick TR. Statistical coil model of the unfolded state: resolving the reconciliation problem. *P Natl Acad Sci USA* **102**, 13099-13104 (2005).

4. Fitzkee N, Rose G. Reassessing random-coil statistics in unfolded proteins. *P Natl Acad Sci USA* **101**, 12497-12502 (2004).

5. Vitalis A, Pappu RV. Methods for Monte Carlo Simulations of Biomacromolecules. *Annu Rep Comput Chem* **5**, 49-76 (2009).

6. Vitalis A, Pappu RV. ABSINTH: A New Continuum Solvation Model for Simulations of Polypeptides in Aqueous Solutions. *J Comput Chem* **30**, 673-699 (2009).

7. Whitford PC, Noel JK, Gosavi S, Schug A, Sanbonmatsu KY, Onuchic JN. An all-atom structure-based potential for proteins: bridging minimal models with all-atom empirical forcefields. *Proteins* **75**, 430-441 (2009).

8. Noel JK*, et al.* SMOG 2: A Versatile Software Package for Generating Structure-Based Models. *PLoS Comput Biol* **12**, e1004794 (2016).

9. Joosten RP*, et al.* A series of PDB related databases for everyday needs. *Nucleic Acids Res* **39**, D411-419 (2011).

10. Tran HT, Wang X, Pappu RV. Reconciling observations of sequence-specific conformational propensities with the generic polymeric behavior of denatured proteins. *Biochemistry* **44**, 11369-11380 (2005).

11. Kohn JE*, et al.* Random-coil behavior and the dimensions of chemically unfolded proteins. *P Natl Acad Sci USA* **102**, 12690-12693 (2004).

12. Anandakrishnan R, Aguilar B, Onufriev AV. H++ 3.0: automating pK prediction and the preparation of biomolecular structures for atomistic molecular modeling and simulations. *Nucleic Acids Res* **40**, W537-541 (2012).

13. Chan CH, Wilbanks CC, Makhatadze GI, Wong KB. Electrostatic contribution of surface charge residues to the stability of a thermophilic protein: benchmarking experimental and predicted pKa values. *PloS one* **7**, e30296 (2012).

14. Lee S, Tikhomirova A, Shalvardjian N, Chalikian TV. Partial molar volumes and adiabatic compressibilities of unfolded protein states. *Biophys Chem* **134**, 185-199 (2008).

Supplementary Table 1

Ultra-High Resolution Protein Set (0.73 - 1.20 Å): First 4 letters are PDB code, fifth letter is chain id. In parenthesis - number of amino acid residues and crystallographic resolution in Å.

2ERL_ (40, 1.00); **1P9GA (**41, 0.84); **1CNR_ (**46, 1.05); **2A26A (**50, 1.20); **1BRFA (**53, 0.95); **2CS7C (**55, 1.20); **1G6XA (**58, 0.86); **1OAIA (**59, 1.00); **2FMAA (**59, 0.85); **2G6FX (**59, 0.92); **1NKD_ (**59, 1.07); **2IGD_ (**61, 1.10); **1G2BA (**62, 1.12); **1V6PA (**62, 0.87); **2SN3_ (**65, 1.20); **1C9OA (**66, 1.17); **1HG7A (**66, 1.15); **1TUKA (**67, 1.12); **1VFYA (**67, 1.15); **2DLBA (**70, 1.20); **2B97A (**71, 0.75); **1WM3A (**72, 1.20); **1WXCB (**72, 1.20); **1CC8A (**73, 1.02); **1I27A (**73, 1.02); **1L9LA (**74, 0.92); **1OK0A (**74, 0.93); **2BWFB (**77, 1.15); **1USMA (**77, 1.20); **1UCRB (**78, 1.20); **1XMKA (**79, 0.97); **1IQZA (**81, 0.92); **1R6JA (**82, 0.73); **1ZZKA (**82, 0.95); **2D8DB (**83, 1.15); **1B0YA (**85, 0.93); **1CTJ_ (**89, 1.10); **1U07A (**90, 1.13); **2BT9A (**90, 0.94); **1X6IB (**91, 1.20); **2FHZB (**93, 1.15); **1C5EA (**95, 1.10); **1LNIB (**96, 1.00); **1CZPA (**98, 1.17); **2AIBA (**98, 1.10); **1NQJA (**98, 1.00); **1KZKB (**99, 1.09); **1MN8D (**100, 1.00); **1PSRB (**100, 1.05); **1M2DA (**101, 1.05); **2DKOB (**103, 1.06); **2H3LA (**103, 1.00); **1LKKA (**105, 1.00); **1TQGA (**105, 0.98); **2GBAA (**105, 0.92); **1M9ZA (**105, 1.05); **2FRGP (**106, 1.19); **1V8HA (**107, 1.20); **1GMXA (**108, 1.10); **1J0PA (**108, 0.91); **2AGYD (**108, 1.10); **1BKRA (**109, 1.10); **1RWYA (**109, 1.05); **2FHZA (**109, 1.15); **1H4XA (**110, 1.16); **1I8OA (**114, 1.15); **2CHHA (**114, 1.00); **1F86A (**115, 1.10); **1SAUA (**115, 1.12); **1O7IA (**119, 1.20); **2ICCA (**119, 1.20); **2F01B (**120, 0.85); **1W0NA (**120, 0.80); **1VR7A (**120, 1.20); **1WN2A (**121, 1.20); **2GUDB (**121, 0.94); **1LWBA (**122, 1.05); **2FWGA (**122, 1.10); **1VL9A (**123, 0.97); **1DY5A (**123, 0.87); **1GU2A (**124, 1.19); **1UNQA (**124, 0.98); **1NWZA (**125, 0.82); **2FJ8A (**125, 1.19); **1VZIA (**126, 1.15); **1JBEA (**126, 1.08); **4LZT_ (**129, 0.95); **1KNLA (**130, 1.20); **1JF8A (**131, 1.12); **1OH0B (**131, 1.10); **1C7KA (**132, 1.00); **1IFC_ (**132, 1.19); **1TU9A (**134, 1.20); **2AXWA (**134, 1.05); **1NKIA (**135, 0.95); **1CZ9A (**139, 1.20); **1RG8A (**146, 1.10); **1EXRA (**148, 1.00); **1A6M_ (**151, 1.00); **1QTNA (**152, 1.20); **1GWMA (**153, 1.15); **2C9VA (**153, 1.07); **1J98A (**153, 1.20); **2FLHB (**155, 1.20); **1UOWA (**159, 1.04); **1Y93A (**159, 1.03); **1P6OB (**161, 1.14); **1L3KA (**163, 1.10); **1TT8A (**164, 1.00); **1WKQA (**164, 1.17); **1N62A (**166, 1.09); **2CE2X (**166, 1.00); **1OBOA (**169, 1.20); **1AMM_ (**174, 1.20); **2AU7A (**175, 1.05); **1EB6A (**177, 1.00); **2C2UA (**178, 1.10); **1I4UA (**181, 1.15); **1WC2A (**181, 1.20); **1KT6A (**183, 1.10); **2AT7X (**184, 0.98); **1PMHX (**185, 1.06); **1QV0A (**185, 1.10); **2BBRA (**189, 1.20); **2PTH_ (**193, 1.20); **2CARA (**196, 1.09); **1QQ4A (**198, 1.20); **1Z0WA (**203, 1.20); **1JM1A (**204, 1.11); **1IXBA (**205, 0.90); **2AB0A (**205, 1.10); **2C71A (**205, 1.05); **1HDOA (**206, 1.15); **1G66A (**207, 0.90); **1H4GB (**207, 1.10); **1KWNA (**207, 1.20); **1SFSA (**213, 1.07); **1ME3A (**215, 1.20); **1K4IA (**216, 0.98); **1W66A (**218, 1.08); **1FYEA (**220, 1.20); **1O08A (**221, 1.20); **2A6ZA (**222, 1.00); **1OLRA (**224, 1.20); **1UAIA (**224, 1.20); **2AWKA (**224, 1.15); **1KG2A (**225, 1.20); **1FSGC (**233, 1.05); **1K7CA (**233, 1.12); **1YMTA (**235, 1.20); **1JBC_ (**237, 1.20); **1GVKB (**240, 0.94); **1QL0A (**241, 1.10); **1HBNC (**248, 1.16); **2J27A (**250, 1.15); **1ZJYA (**251, 1.05); **1QXYA (**252, 1.04); **1XQOA (**256, 1.03); **1MOOA (**256, 1.05); **1P1XA (**260, 0.99); **1UWCA (**261, 1.08); **1NYMA (**263, 1.20); **1ARB_ (**263, 1.20); **1XDNA (**265, 1.20); **1WUIS (**267, 1.04); **1KQPA (**271, 1.03); **2CI1A (**273, 1.08); **1WXCA (**273, 1.20); **1IC6A (**279, 0.98); **2BOGX (**280, 1.04); **1E9GB (**283, 1.15); **1QTWA (**285, 1.02); **1LC0A (**290, 1.20); **2EUTA (**291, 1.12); **1RTQA (**291, 0.95); **2J45B (**297, 1.14); **2CIWA (**298, 1.15); **2BLNA (**298, 1.20); **8A3HA (**300, 0.97); **2CNQA (**301, 1.00); **1V0LA (**302, 0.98); **1ZL0B (**303, 1.10); **1Z2NX (**311, 1.20); **2IAVA (**312, 1.07); **1T2DA (**315, 1.10); **1PWMA (**316, 0.92); **1YS1X (**320, 1.10); **1DS1A (**323, 1.08); **1RYOA (**324, 1.20); **1OEWA (**328, 0.90); **2BW4A (**334, 0.90); **2C1VA (**335, 1.20); **1YFQA (**342, 1.10); **1YQSA (**345, 1.05); **1M15A (**356, 1.20); **1C0PA (**363, 1.20); **1VYRA (**363, 0.90); **1GA6A (**369, 1.00); **1N8KA (**374, 1.13); **3SIL_ (**379, 1.05); **1KJQB (**385,

1.05); **1MUWA (**386, 0.86); **1RA0A (**423, 1.12); **1UG6A (**426, 0.99); **2BMOA (**437, 1.20); **1HBNB (**442, 1.16); **2BF6A (**449, 0.97); **1M1NA (**477, 1.16); **2FBAA (**492, 1.10); **1QW9A (**497, 1.20); **1GWEA (**498, 0.88); **1JETA (**517, 1.20); **1M1NB (**522, 1.16); **1Q6ZA (**524, 1.00); **1WUIL (**532, 1.04); **1HBNA (**543, 1.16); **1UWKB (**553, 1.19); **2BHUA (**580, 1.10); **1SU8A (**633, 1.10); **1N62B (**804, 1.09); **1QWNA (**1014, 1.20).

Supplementary Table 2

Volumes of Ionization for Various protein Groups

**Protein Group**	**Volume (Å^3)[1]**
N terminus	-8.0
Arg	-9.1
Lys	-9.8
His	-2.3
Asp	-20.1
Glu	-19.6
C terminus	-17.4

[1] Volume changes are for the reactions $AH \rightarrow A^{-} + H^{+}$ and $B + H^{+} \rightarrow BH^{+}$ where A is the acid and B is the base. The volumes of ionization can be used to predict the volumes of a protein in the native or unfolded state by adding the following term to equation 5 in the main text:

$$V_{el} = f_{Nter} \cdot V_{Nter} + f_{Cter} \cdot V_{Cter} + \sum_{i=D,E,R,K,H} f_i \cdot N_i \cdot V_i$$

where N_i is the number of a given type of ionizable group, f_i is fraction exposed, and V_i is the ionization volume [14].
